# Spectroscopic Ellipsometry and Quartz Crystal Microbalance with Dissipation for the Assessment of Polymer Layers and for the Application in Biosensing

**DOI:** 10.3390/polym14051056

**Published:** 2022-03-07

**Authors:** Ieva Plikusiene, Vincentas Maciulis, Arunas Ramanavicius, Almira Ramanaviciene

**Affiliations:** 1 Nanotechnas–Center of Nanotechnology and Materials Science, Faculty of Chemistry and Geosciences, Vilnius University, Naugarduko Str. 24, LT-03225 Vilnius, Lithuania; vincentas.maciulis@ftmc.lt (V.M.); arunas.ramanavicius@chf.vu.lt (A.R.); 2 State Research Institute Centre for Physical Sciences and Technology, Sauletekio Ave. 3, LT-10257 Vilnius, Lithuania; 3 Department of Immunology, State Research Institute Centre for Innovative Medicine, Santariskiu g. 5, LT-08406 Vilnius, Lithuania

**Keywords:** spectroscopic ellipsometry, quartz crystal microbalance with dissipation (QCM-D), characterization of polymer layers, application for biosensing, immunosensors, biosensors, conducting polymers, molecularly imprinted polymers (MIPs)

## Abstract

Polymers represent materials that are applied in almost all areas of modern life, therefore, the characterization of polymer layers using different methods is of great importance. In this review, the main attention is dedicated to the non-invasive and label-free optical and acoustic methods, namely spectroscopic ellipsometry (SE) and quartz crystal microbalance with dissipation (QCM-D). The specific advantages of these techniques applied for in situ monitoring of polymer layer formation and characterization, biomolecule immobilization, and registration of specific interactions were summarized and discussed. In addition, the exceptional benefits and future perspectives of combined spectroscopic ellipsometry and QCM-D (SE/QCM-D) in one measurement are overviewed. Recent advances in the discussed area allow us to conclude that especially significant breakthroughs are foreseen in the complementary application of both QCM-D and SE techniques for the investigation of polymer structure and assessment of the interaction between biomolecules such as antigens and antibodies, receptors and ligands, and complementary DNA strands.

## 1. Introduction

Polymers, whether natural or artificial, have unique properties depending on the type of molecules, which form the polymer backbone, and inter-chain interactions. Polymer layers might exhibit properties such as: mechanical strength, toughness, resilience, flexibility toward stretching, electrical conductivity, and optical transparency. Moreover, polymer layers can be thin or thick, evenly distributed on the surface, or randomly dispersed [1]. Different methods and conditions are employed for the synthesis of polymers with desired properties. If required, entrapment of some nanomaterials can improve the properties of polymers [2,3,4,5]. All of this leads to a wide range of practical polymer applications. Polymers are used in almost all areas of our modern life, including various analytical techniques. Polymeric films play an important role in the design of chemical and biological sensors [6,7]. The immobilization of biomolecules or formation of molecularly imprinted polymers (MIPs) at interfaces using various polymers films represents an extremely important task in the design and development of biosensors [8,9,10,11,12]. For this purpose, analytical methods that can provide information about the physical and chemical properties of polymeric films are required. Polymers can be characterized by chromatographic, spectroscopic, microscopic, thermal, rheometric, mechanical, optical and other methods [13]. Optical and acoustic methods are widely used for noninvasive, label-free, in situ monitoring of polymeric film formation, protein immobilization, registration of specific interactions, and analysis [14]. The label-free, ultrasensitive, and high signal-to-noise ratio optical sensing approach named electrochromic optical recording (ECORE) was successfully applied for the recording of long-term bioelectric potentials of cardiomyocytes, neurons, and brain slices [15,16]. However, two techniques based on the surface-sensitive methods that bear different operating principles attracted special attention. One of them is optical spectroscopic ellipsometry (SE), and the second is an acoustic quartz crystal microbalance with dissipation (QCM-D).

SE isa non-destructive, non-invasive optical technique that enables the measurement of changes in the light polarization state after reflection from or transmission through the sample. The concept ‘ellipsometry’ originates from the practice where polarized light is usually elliptically polarized upon reflection of the sample [17]. The vigor of ellipsometry is that it measures the change in polarized light upon reflection or transmission by the sample but not the absolute intensity [18]. Measurement of changes in light polarization state renders ellipsometry an attractive tool for the investigation of polymers thin film optical properties [19]. Such information including sample refractive index, extinction coefficient, surface roughness, and thickness of the layer can be extracted from ellipsometric measurements. Compared to others SE represents the most general reflection-based technique as it can be employed to determine all information that is possible with any other reflection based method [20]. The sensitivity of SE can be maximized by applying the required incident angle of light which depends on the optical constants of the sample [17].

QCM-D is an analytical technique sensitive to changes in surface mass and viscoelasticity, resulting from processes occurring on surfaces or within thin films [21]. Various biosensors and biosensing systems based on QCM-D readout are becoming increasingly attractive for the detection of chemical and biological molecules [22]. This technique is a powerful tool for measuring the real-time kinetics of immobilization and interaction between biomolecules [23]. Changes in the resonant frequency (Δ*f*) of the oscillating quartz crystal are induced by the change in surface mass (Δ*m*) [24]. Energy damping (Δ*D*), so called dissipation of the oscillating quartz crystal, is observed when the formed layer changes its viscoelastic or structural properties [23,25,26]. The measurement of Δ*f* and Δ*D* using QCM-D technique enables simultaneous real-time monitoring of both parameters. These measurements can provide information on the thickness of the effective layer, the conformational and viscoelastic properties, and the hydration state of the polymeric layer [27,28].

SE and QCM-D are both highly surface-sensitive and can be used to study various polymer films ranging in thickness from angstroms to micrometers [29]. As a result, the parameters of polymeric materials, such as adsorbed amounts of polymer, film thickness, optical and viscoelastic properties, can be extracted from the measured characteristics as quantitative information [14,26,30]. Furthermore, both techniques can be combined simultaneously during the same measurement and provide information about the real mass of deposited material.

## 2. Operation Principle of Spectroscopic Ellipsometry

**Figure 1:**
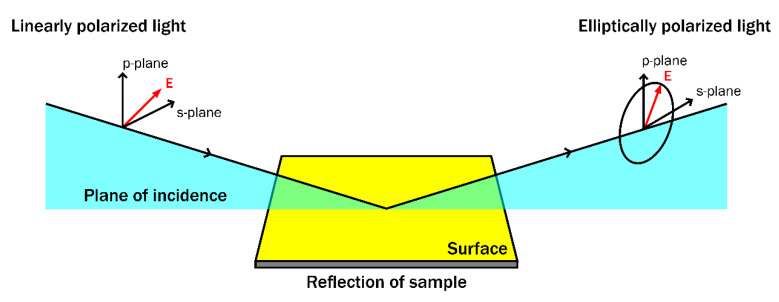
Operation principle of spectroscopic ellipsometry.

Due to its non-contact, non-destructive, and high precision nature, SE can be successfully applied to various bulk materials and thin film analysis, including the wide range of polymers and in situ studies of biomolecule interactions and/or their adsorption on different surfaces [9,31,32,33,34,35,36]. The general operating principle of SE is presented in Figure 1.

This optical technique uses polarized light for the measurement of the optical properties of various thin films described by the complex refractive index (*N*). The *N* refers to the refractive index (*n*) and the extinction coefficient (*k*) and represents the real and imaginary parts of the complex refractive index [18]. The refractive index denoted by *n*, is the ratio of the speed of light in a vacuum and in the material. The *k* is related to the decrease of material’s optical absorption [17,18]:

(1) N=n+ik 

For transparent bulk materials samples or thin films, the *k* values are negligible, due to the high penetration depths of light. Therefore, for transparent materials, the values of *k* are very close to zero, thus in this case *N* = *n* [18]. For the absorbing materials, *n* and *k* are not constants and depend on the wavelength of light (*λ*). Using ellipsometry, known polarization light is reflected from or transmitted through the sample at a defined angle of incidence. The form of the ellipse can be measured by a detector, and data processing can relate this measurement to the ellipsometric parameters *Ψ* and Δ [37]. After light reflection/transmission from the sample the change in polarization state is measured, obtaining two so-called ellipsometric parameters *Ψ* and Δ. The ellipsometric parameter *Ψ* corresponds to the amplitudes ratio of the polarized light wave upon reflection and Δ corresponds to the phase difference of the light wave between the *p*- and *s*- polarization [17]. These ellipsometric parameters are measured as the ratio of the light wave in-plane (*p*-) and out of plane (*s*-) polarizations reflection described by Fresnel’s formula from which the main ellipsometry equation describing the complex reflection ratio is derived [17,37,38]:

(2) $$\rho =\frac{r_{p}}{r_{s}}=tan\left(\Psi \right)e^{i\Delta }$$

**Figure 2:**
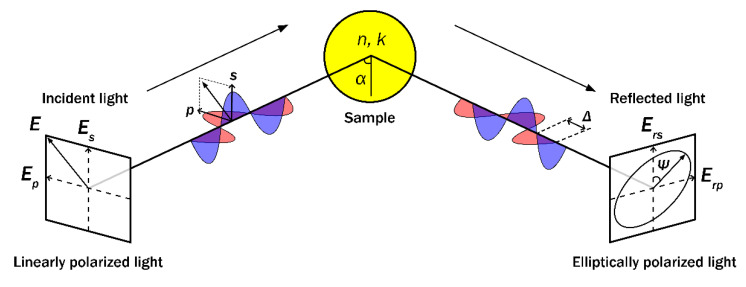
Schematic representation of ellipsometric measurements. *E*–electric field vector, *E*_s_–component of electric field vector perpendicular to the plane of incidence *E*_p_–component of electric field vector parallel to the plane of incidence, *n*–refractive index of the sample, *k*–extinction coefficient of the sample, *E*_rs_, *E*_rp_–perpendicular and parallel reflected field amplitudes. *Ψ*–ellipsometric parameter that corresponds to amplitudes ratio upon reflection, Δ–ellipsometric parameter that corresponds to phase difference between *p*- and *s*- polarizations, α- angle of incident light.

Here *ρ* is the complex reflection ratio of the *p*-polarized (*r_p_*) and *s*-polarized (*r_s_*) light waves. The schematic representation of ellipsometric measurement is presented in Figure 2. 

Due to the complex reflection ratio measurements, ellipsometry does not require reference measurements, and this renders it a very attractive technique that can be applied for analysis of different thin films. Additionally, this makes ellipsometry a precise tool for the determination of some physical properties (e.g., dielectric constants) of bulk materials and thicknesses of various thin films.

## 3. Limitations of Spectroscopic Ellipsometry

Despite the number of advantages SE contains some drawbacks. The main disadvantage of SE is that in order to determine information such as thicknesses and optical constants of the polymer or any other layers, a numerical optical model must be created and fitted to the experimentally obtained optical data. An optical model describes the optical constants of the polymer (or other) sample and its thickness. The construction of an optical model requires special knowledge in optics, mathematics, chemistry, etc. If the sample is very thick and can be evaluated as a bulk material and isotropic, the optical model is not required. However, ellipsometry in many cases is applied for thin film investigation, thus usually the unique optical model must be created and adapted. The surface roughness of the measured samples must be low and not exceed 30% of the sample layer thickness, and the measurement must be carried out at an oblique incidence. When surface roughness is high, it reduces the intensity of reflected light, and ellipsometric measurement becomes inaccurate, since ellipsometry evaluates a polarization state from the registered signal of reflected or transmitted light [17]. When the thickness of the layer is measured by SE, it has to be set in a way that the light beam that travels through the layer has to return to the surface. If the layer is absorbing, thickness measurements become limited to thin, semi-opaque films. This can be avoided by measuring SE only in the particular spectral range where the layer is transparent or its absorption is low. SE technique is mainly applied for investigation of the layers with thicknesses ranging from a few nanometers to a few microns. If the layer thickness is higher than several tens of microns, the interference oscillations become problematic and renders it difficult to resolve. In this case, another measurement technique should be applied [39]. SE can be used in different modes, such as reflection, transmission, and in situ, and it can be applied for various needs, including real-time biosensing of different analytes.

## 4. Spectroscopic Ellipsometry for Polymers Analysis

### 4.1. The Application of Spectroscopic Ellipsometry for the Characterization of Optical Properties of Polymers

The *N* of some polymers has been measured using SE due to its high precision and non-destructive nature [40,41,42]. Thin films of poly(9,9-di-*n*-octylfluorene-*alt*-benzothiadiazole) polymers exhibited anisotropic optical constants with larger in-plane values of the *n* and *k* in comparison to out-of-plane values. In this work, authors applied variable angle SE and obtained anisotropic optical constants of thin film polymers of different molecular weights in the pristine and annealed states [40]. It is difficult to determine the index of refraction for very thin polymer films with thicknesses smaller than 10 nm [42,43]. However, there is an exception when SE is applied for this purpose. The high sensitivity of SE and effects of incident light angle and wavelength can be successfully applied for the evaluation of optical constants and thicknesses of very thin polymer films (down to 5 nm) [42]. The radiative properties of polymers are very important for the design of radiative cooling systems. However, the precise knowledge of polymer complex refractive indices is still undergoing research. SE can be considered as the most accurate technique for measuring complex refractive indices of materials, including polymers with strong absorption. This technique was applied for the measurement of complex *N* of seven polymers, such as polydimethylsiloxane (PDMS), polymethyl methacrylate (PMMA), polycarbonate (PC), polystyrene (PS), polyethylene terephthalate (PET), polyvinyl chloride (PVC) and polyetherimide (PEI) in combination with the ray tracing method (RTM) from the near-infrared to mid-infrared bands (2–20 μm) [44]. Moreover, SE was applied for the studies of electrochemically synthesized poly(ortho-phenylenediamine) (PoPD) film with MIPs used for the detection of the main aviation biokerosene contaminant hexahydrofarnesol. SE was employed to obtain the thickness of the PoPD film and for investigation of the optical and dielectric properties of this material [45]. The optical properties of thin polymers films containing azo compounds in the side chain are important for various applications, such as optical storage, optical switching, optical limiting, signal processing, optical modulators, holography, and nonlinear optical devices, as well as various types of photonic devices [46,47,48,49,50,51]. Such optical properties as absorption coefficient, refractive index and energy band gap of polymers with heterocyclic azo dyes in the side-chain were investigated using SE combined with transmittance measurements [46]. Poly (3-hexylthiophene), which is an organic semiconductor, and the fullerene derivative (6,6)-phenyl-C61-butyric acid methyl ester are well studied organic materials with wide applications in organic electronics, especially in organic photovoltaic devices. Undoubtedly, the knowledge regarding the optical constants of materials is crucial for the design of the organic photovoltaic devices, as such devices usually have sandwich architecture. For this purpose, variable temperature SE was applied, which provided information about *n*, *k*, and thickness of the layer dependence on the temperature *T* during the investigation of phase transition [52,53,54,55]. This feature of SE was widely applied studying polymers such as polystyrene (PS) [56], poly(α-methylstyrene) (PαMS) [57], poly(methyl methacrylate) (PMMA) [58,59], polyfluorenes (PF) [60], quinoxaline and pyridopyrazine-based polymers [61], and conjugated polymers [29,62]. 

### 4.2. In Situ Spectroscopic Ellipsometry Application for the Determination of Polymers Thickness and Optical Properties 

Since SE is well-known as a non-invasive, non-destructive optical technique, it can be successfully applied for real-time monitoring of polymer optical properties. In situ SE allows information regarding the changes of the refractive index and thickness of the polymer layer to be obtained. It has been successfully used to study polymer infiltration. Sequential infiltration synthesis (SIS) of alumina using trimethylaluminum (TMA) and H_2_O was studied using a series of polymers. Different thicknesses of PMMA and PS thin films infiltration were investigated using time resolved SE. Different swelling behaviors of the PMMA and PS layers were obtained. The larger solubility of TMA in PMMA in comparison to PS was observed. The results showed that formed Al_2_O_3_ film was thicker and denser for the PMMA layer with respect to the PS layer, and PMMA layer thickness increased after 10 SIS cycles [63]. In situ SE was applied for the analysis of thin polyimide (PI) films formed on the surface of the silicon wafer in various buffer solutions. The results obtained showed that the stability of PI is poor in aqueous solutions and PI cannot be applied for biosensing as a result of its swelling. However, TiO_2_ films of 50 nm thickness obtained using atomic layer deposition (ALD) technique are very promising for the protection of PI [64]. In situ infrared spectroscopic ellipsometry (IRSE) is a powerful tool for the characterization of structural and chemical properties of polymer brushes during stimuli-induced switching experiments [65]. IRSE and SE are highly sensitive nondestructive techniques that allow protein adsorption to be studied on polymer brushes in a liquid environment. Moreover, using IRSE, it is possible to investigate how changes in pH and temperature affect such adsorption. An external stimulus such as pH or temperature can provide such polymer brushes with attractive properties for surface application in biosensing. Polymer brushes are thin films of polymer molecules covalently attached to the substrate and stretched due to excluded volume interactions. One of such polymers studied using IRSE was poly(N-isopropylacrylamide) (PNIPAM). In this study IRSE was used to characterize the 12.6 nm polymer brush from the temperature-sensitive swelling behavior of PNIPAM. The results obtained provided information on the thickness of the brush as well as the water content inside the brush [66]. A binary polymer brush consisting of weak polyelectrolytes poly(ethylene glycol) (PEG) and poly(acrylic acid) b-poly(styrene) (PAA-b-PS) in a 50/50 composition was investigated with real-time infrared synchrotron mapping ellipsometry in different pH aqueous solutions. IR spectral analysis identified the chemical structure of previously mentioned polymers after injection of the solution having defined pH [67]. Polymer-like hydrogenated amorphous carbon films were also studied in situ by IRSE. Dielectric constant and vibrational frequencies, widths and intensities as a function of macroscopic properties were obtained from the here mentioned polymer-like films [68].

### 4.3. Application of Spectroscopic Ellipsometry for the Assessment of Polymers Used in Biosensing 

Ellipsometry in total internal reflection mode (TIRE) is a powerful tool for applications in biosensing [32,33,69,70]. In many cases during the application of TIRE, the high sensitivity of SE is combined with that of the surface plasmon resonance (SPR). This provides the possibility of registering two kinetic curves simultaneously instead of the single curve registered by SPR-based biosensors [33]. The simultaneous measurement of two kinetic curves provides the ability to obtain information on changes in intensity and phase that result in more accurate refractive index and thickness calculations and provides more information on the interaction of biomolecules at the solid–liquid interface [71]. Due to this, the sensitivity of TIRE is nearly 10 times higher than that of standard reflection techniques, including SPR. Phase shift measurements make the TIRE method a powerful tool for the detection of biomolecules at the solid–liquid interface, proving even better than widely applied SPR. The sharp phase jump under total internal reflection (TIR) conditions provides higher sensitivity to refractive index changes due to the excitation of evanescent surface waves that exhibit exponential decay [72,73,74]. When phase shift measurements under TIR conditions are used for biosensing, its sensitivity overcomes typically applied intensity techniques such as SPR. A very high phase shift in TIRE is achieved due to ellipsometric parameter Δ dependence on the incident angle of the light under TIR conditions and the changes of *n* and thickness in the wavelength range near the SPR minima during the formation of a biolayer [73,75]. The ECORE method was also successfully utilized in TIR mode for the optical recording of label-free neuroelectric activities of cardiomyocytes, neurons, and brain slices using the electrochromism phenomenon of poly(3,4-ethylenedioxythiophene) polystyrene sulfonate (PEDOT:PSS) thin films. The optical absorption of PEDOD:PSS layers can be modulated by an applied voltage [15]. The zeonor (ZR), a type of cycloolefin polymer, has received interest in its application for diagnostics owing to its excellent optical and chemical properties and good machinability. Thus, TIRE was applied to monitor the single stranded DNA (ssDNA) hybridization with the complementary strand and to detect human chorionic gonadotrophin (hCG) using sandwich immunoassay format on ZR surface functionalized with carboxyl groups. Furthermore, modeling of the ellipsometric spectra and further calculation allowed the authors to evaluate the surface mass density of the ssDNA or capture anti-α-hCG antibodies bound to the ZR surface. It was discovered that only a small thickness change of 0.25 ± 0.12 nm after aminated Sa19 ssDNA binding to carboxyl groups modified ZR surface without the activation of functional groups was registered during the negative control experiment. For the positive control experiment, the effective calculated thicknesses of both captured ssDNA and formed double-stranded DNA (dsDNA) were 2.02 ± 0.16 nm and 3.49 ± 0.42 nm, respectively. Such results showed that the hybridization increased the effective thickness, suggesting that the capture ssDNA could form a layer composed of coiled oligonucleotides that can be stretched during the hybridization with the complementary ssDNA. Additionally, the ZR surface modified with carboxyl groups was used for the development of hCG sandwich immunoassays. Following the covalent immobilization of the capture anti-α-hCG antibody, a large spectral shift of ellipsometric parameters was observed. This result showed that the surface mass density of the anti-α-hCG antibodies increased with an increasing concentration of antibodies in the solution during the initial surface functionalization step. The subsequent binding of 2 μg mL^−1^ concentration hCG to the anti-α-hCG antibody was followed by another shift of ellipsometric parameters. In the final stage, second detection anti-β-hCG antibody binding was investigated. However, the final changes in the *Ψ* and Δ spectra for binding of hCG and anti-β-hCG antibodies were small and not particularly distinctive in comparison to shifts obtained for anti-α-hCG immobilization [76]. The polyacrylamide (PAAm) is a water-soluble polymer and carries positive charges between pH 4.8 and pH 7.4, providing electrostatic interactions with DNA molecules. SE was applied for the evaluation of the silicon wafer surfaces modification by PAAm, and further dsDNA layer formation on the amino terminated surface. First, three silicon surfaces modified with PAAm were examined by SE. The different surface morphology and thicknesses, depending on the polymerization time (after 8 h, 16 h, and 24 h), were obtained. The thickness of the formed PAAm layer after 8 h was 6.80 nm, while after 16 h it was 8.14 nm. Highest surface coverage with PAAm was reached after 24 h due to the increasing molecular weight of the PAAm chains on the surface, and the thickness was 9.18 nm. Moreover, in this work, it was shown that the thickness of the overlayer increases with a higher concentration of dsDNA (0.065, 0.12, and 0.25 mg mL^−1^) and incubation time (5, 8, and 12 h). The thickness of the immobilized dsDNA layer on the polymer surfaces measured 9.82 nm after the first 5 h, and increased to around 12.85 nm after 12 h [77]. The protein monolayers conformational stability after their immobilization is very important for further application in biosensing. Plasma polymers became attractive for protein immobilization owing to the simplicity of the method and large-scale capability. The immobilization of enzymes in plasma polymers and their stability was investigated using SE. Immobilized tetrameric bovine liver catalase was more stable than the monomeric horseradish peroxidase (HRP) at the surface/air interface [78]. In another work, HRP and anti-HRP antibody interaction was investigated. The nitrogen-containing plasma polymers (NPPs) were used to design three biosensors based on single-wavelength ellipsometry at fixed incident angle. Spectra of ellipsometric parameter *Ψ* were calculated for three sensors based on NPPs layers of different thicknesses (50, 70, 90 nm) formed on a 150 nm silicon nitride coated silicon wafer surface. It was discovered that the sensitivity of *Ψ* parameter near ‘valley minima’ was low and varied significantly with small changes in the wavelength applied for the measurement [79]. The summary of SE and QCM-D application for the assessment of polymer layers and for the application in biosensing is presented in Table 1.

### 4.4. Application of Imaging Ellipsometry for the Analysis of Polymers 

The difference in imaging ellipsometry (IE) from standard reflection ellipsometry is that it is combined with optical microscopy. Compared to conventional ellipsometry, IE provides the advantage of spatially high vertical resolution and also bears a large field of view. Method of IE can be applied for visualization of the layer thickness distribution on a large surface area [101]. Various biological analytes can be detected using specific ligands covalently immobilized on the silicon wafers with 3-aminopropyltriethoxysilane (APTES) additionally chemically modified by saturated succinic anhydride solution to obtain carboxyl groups. Thus, a biosensor based on the IE and incorporating microfluidic system was developed for the detection of hepatitis B virus (HBV) infection markers. The results showed that such a biosensor can simultaneously detect up to five markers within 1 h and the obtained results were in good agreement with ELISA results. The reported cutoff values (optimal decision thresholds) for HBsAg, anti-HBs, hepatitis B e antigen, hepatitis B e antibody and core antibody were as follows: 15%, 18%, 15%, 20% and 15%, respectively [102]. The cutoff value of each marker was analyzed by referring to receiver operating characteristic curve. The best cutoff exhibits the highest true positive rate together with the lowest false positive rate. The application of APTES and glutaraldehyde (APTES-Glu) for the modification of the silicon surface and further covalent immobilization of proteins for immunoassay was investigated by IE. This investigation resulted in the higher density and stability of human IgG antibody layer on the silicon surface modified with APTES-Glu than that modified with dichlorodimethylsilane (DDS). Desorption of 10% of the human IgG from the DDS modified surface was observed; moreover, the IgG immobilized on the APTES-Glu surface bounded more anti-IgG molecules than that on the DDS surface [103]. Total internal reflection imaging ellipsometry (TIRIE) was applied for the investigation of giant lipid vesicles (GUVs) interaction with surface modified by poly-L-lysine. This investigation showed that TIRIE can be successfully applied in the design of biosensors for the detection of GUVs adsorption, desorption, or flattering on the PLL coating. Two concentrations of PLL solutions were used for the coating of the sensing surface: c_0_ = 1 mg mL ^−1^ and 1:100 dilution c_0_/100. The adhesion of GUVs was studied using three different pH levels (pH = 3.1, 7.0, and 10.0) of the suspending glucose solution. The percentage of intact adhered GUVs increased with increasing pH, the highest percentage around 75% was reached using 100 times diluted PLL at pH = 10.0 [104]. In addition, the hydration of biopolymers was investigated using IE. Thickness dependence on the osmotic pressures within the hydrated films was quantitatively studied employing this method by measuring equilibrium film thickness as a function of relative atmospheric humidity. The hydration of cellulose films formed by regeneration of trimethylsilylcellulose films deposited on silicon wafers by the Langmuir–Blodgett method was investigated and compared with that determined for cellulose films produced using spin-coating method. It was found that the initial films thicknesses values (24–104 Å) were proportional to the number of monolayers (6–20 monolayers), and that the monolayer thickness was in the range from 4.0 to 5.5 Å. The film thickness changes were small when the humidity was below 80% but a significant increase was observed at higher humidity conditions. The maximal thickness of the film produced by spin coating after regeneration was 182 Å, and showed the highest swelling ratio [105].

## 5. Quartz Crystal Microbalance with Dissipation for the Assessment of Polymers

Both methods SE and QCM-D are surface sensitive and suitable for the analysis of polymer layer formation or application for biosensing at the solid–liquid interface. The different operating principles of these techniques are highly complementary and their combination can provide information on the optical and mechanical properties of polymer layers in the same measurement [14]. While ellipsometry describes optical properties of the polymer layers, the QCM-D can show mechanical properties such as elasticity and viscosity. Furthermore, the operating principle of QCM-D and its application for polymer investigations are reviewed. 

### 5.1. Operation Principle of Quartz Crystal Microbalance with Dissipation 

**Figure 3:**
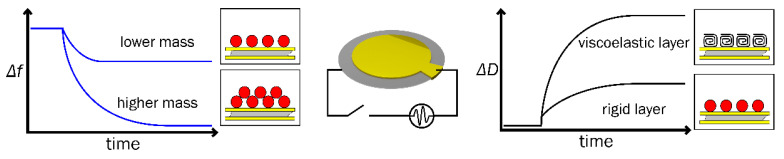
Schematic representation of QCM-D measurement and operating principle. Δ*f*–time depended change in frequency, Δ*D*–time depended change in energy dissipation.

Both QCM and QCM-D are powerful acoustic shear-mode techniques that can be applied to the measurements of real mass changes and viscosity during the formation of the polymer layer at the solid–liquid interface. This method is capable of detecting extremely small changes (at nanoscale resolution) in the chemical, mechanical, and electrical properties of layers. These properties are very important in studying the dynamic processes of immobilization or adhesion of the molecule to the surface, the interaction of the formed layer with the analyte, and during the swelling or collapsing of the layer [21]. Quartz crystal resonators are applied in QCM-D devices, while a characteristic frequency of mechanical oscillations is generated on the crystal by applying an alternating current [21]. A quartz crystal resonator based on an ‘AT-cut’ quartz crystal of piezoelectric material with deposited metal electrodes is employed in the QCM-D setup. An ‘AT-cut’ quartz crystal oscillates at a defined fundamental frequency (e.g., 5 MHz for some commercially available QCM resonators) by applying an alternating current, and generates a standing shear acoustic wave which propagates perpendicularly to the crystal surface. The molecules can adsorb or desorb from the sensor disk surface and, the frequency of oscillation is highly sensitive to the mass changes at the sensor surface. The resonance frequency of the sensor decreases as more mass is added due to increased inertia [106]. The schematic representation of the measurement and operating principle of QCM-D is presented in Figure 3. Additionally, the thickness of the crystal and its resonant frequency influence the sensitivity of the sensor. Regular QCM measures only Δ*f* and the surface mass density is evaluated using the Sauerbrey equation, while QCM-D measures both Δ*f* and Δ*D* and provides information not only regarding the surface mass but also the viscoelastic properties of the layer. Regular QCM was successfully applied for the evaluation of the enzymatic synthesis of conducting polymer Ppy layer on the surface of the premodified sensor disk with GOx over time [86], and for the evaluation of the electrochemical formation of the aggregated Ppy particle-based layer [87]. The Δ*D* is another characteristic that is additionally registered, while QCM-D measurements are obtained. The introduction liquids with different physical properties, such as density and viscosity, can be used to extract information relating to surface coverage and spatial orientation of adsorbed molecules [107]. Thus, during measurement QCM-D provides two characteristics: Δ*f*, that gives quantitative information about the adsorbed mass and Δ*D*, that provides information about the viscoelastic properties of the polymeric layer formed on the surface of the piezoelectric quartz sensor. Both characteristics are measured simultaneously. Measurements of mass and viscosity are the most common applications of QCM-D. 

Application of an alternating current by the electrodes causes an in-plane shear-mode oscillation of the piezoelectric quartz crystal [108,109]. The resonant frequency harmonics of the quartz crystal oscillations are affected by the mass per unit area at the crystal surface. This dependence is described by the Sauerbrey equation [24]:

(3) $$\Delta m=-\frac{C}{n}\Delta f_{n}\;$$

where Δ*f_n_* is the change in resonance frequency at the n^th^ harmonics (n = 1, 3, 5…), Δ*m* is the mass deposited per unit area of crystal surface, and *C* is the mass sensitivity constant of the instrument. For a 5 MHz crystal, *C* is 17.7 ng∙Hz^−1^∙cm^−2^ [108].

The Sauerbrey equation can be applied for the characterization of the formed layer on the quartz crystal disk surface, but this equation provides solid results only in the case where the formed layer is rigid, homogeneous, and evenly distributed over the surface and bears a well-defined smooth interface with bulk liquid or in air/vacuum. However, this equation contains limitations for application because the Sauerbrey relation becomes inappropriate when the layer formed on the resonator disk surface is soft, rough, and heterogeneous, which usually happens when the measurements of rather large biomolecules (e.g., proteins, DNA) are performed in liquids. This causes not only changes in mass, but also Δ*D* changes of the quartz crystal due to the viscoelastic properties of the formed layer [28]. The *D* is described using the ratio between the energy dissipated during one oscillation cycle (*E_d_*) and the energy stored in the oscillating crystal (*E_s_*) using the following equation [108]:

(4) $$D=\frac{E_{d}}{2\pi E_{s}}$$

However, if the viscoelastic properties of the layer are minuscular, the film may still be swollen due to the effect of the solvent. Solvation cannot be fully determined solely by QCM-D measurements in liquid, this requires QCM-D measurements in dry and wet conditions, which may not be possible. But for dry conditions measurements, SE can be applied. Solvation can be calculated from the combination of QCM-D data with SE since the contribution of solvent to optical density is much lower than the influence on the QCM signal [110]. 

### 5.2. Application of Polymers in QCM-D-Based Biosensors

Several different polymers, including π-π conjugated polymers, such as Ppy, have been used in the fabrication of QCM-D-based biosensors. The optical properties of conductive polymers can be employed as a signal for the investigation of biochemical reactions [111,112]. Polymers such as poly(3,4-ethylenedioxythiophene) (PEDOT) and 3,4-ethylenedioxythiophene (EDOT) were also successfully applied for biosensing. EDOT derivatives were applied in biosensors design to link GOx to the electrode surface with further application for amperometric glucose detection [113]. The growth of PEDOT films from vapor phase was investigated using QCM-D. The obtained results showed that the kinetics of polymerization and the viscoelastic properties of the films varied and four distinct stages in film growth were observed [114]. PEDOT electrodeposited on platinum electrodes doped with the laminin peptides DEDEDYFQRYLI and DCDPGYIGSR were used for cell attachment [115]. Multilayer films formed from PAA, PAH, and silica nanoparticles were used as templates for the preparation of thin nanoporous polymer films that were further applied for the adsorption of bovine serum albumin (BSA). QCM was applied to investigate the growth of multilayers with different size silica nanoparticles ranging from 25 to 85 nm and at different pH levels. The obtained information showed that the amount of adsorbed BSA on the porous thin films increased with increasing bilayer number. Thus, the successful infiltration of protein in the films was confirmed by QCM-based detection [82]. Ochratoxin A (OTA) is a highly toxic compound that contaminates a wide variety of foods, including grapes, coffee, wheat, and other cereal crops. Due to its presence in the food chain, it imposes a hazard on both human and animal health. This explains why fast OTA detection is very important. For this purpose, an indirect competitive, rapid, and sensitive QCM-D-based biosensor was developed for the detection of OTA using antibodies that specifically recognize analyte in the solution. In order to determine OTA in the wine avoiding nonspecific adsorption on the surface of polyphenols, poly(vinylpyrrolidone) (PVP) was mixed with the sample [22]. QCM-D-based immunosensor for bovine leukemia virus antigen gp51 detection was successfully developed using sensor disk modified with gold nanoparticles adsorbed on positively charged PLL, and modified with reduced antibody fragments via thiolate bonds. Furthermore, the specific antibody and antigen interaction was compared with the nonspecific interaction of gp51 with BSA modified surfaces by employing ΔD/Δf plots [92]. Human serum albumin (HSA) and pseudorabies virus antigen were immobilized via entrapment in the polymer layer on the surface of the QCM sensor disk during the electrochemical synthesis of Ppy. In this way, the modified sensor disk showed a higher frequency shift after interaction with specific antibodies than using the conventional physical immobilization method of antigens [85].

**Figure 4:**
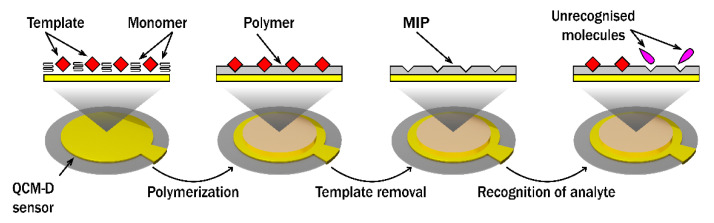
Preparation and working principle of molecularly imprinted polymer (MIP) on a QCM-D sensor disk.

The growing demand for analytical instruments that are highly sensitive, fast, and stable to detect a variety of analytes has led to the development of new sensors and biosensors based on a variety of polymers. Extensive research has been carried out on the preparation and application of MIPs as analyte recognition materials due to their high selectivity and affinity, long stability, resistance to pressure, high temperatures, and extreme pH [6]. The preparation of MIP and the recognition of the analyte principle is presented in Figure 4. MIP technology enables the recognition of different sized molecules, such as caffeine [10,116], uric acid [117], DNA [8], enzymes [118], proteins [119] or whole cells [120]. MIP produced using dopamine D1 receptor (D1R) as a template was produced on a sensor disk for QCM-based assessment on the binding activity of agonists and antagonists of D1R. The mentioned receptor was imprinted into an oligomer film consisting of acrylic acid: N-vinylpyrrolidone: N,N’-(1,2-dihydroxyethylene) bis-acrylamide in a ratio of 2:3:12. The prepared MIP can distinguish free receptor and receptor–dopamine complexes, as well as receptor–antagonist complexes [81]. Direct detection of dengue virus protein NS_1_ on the surface of MIP-modified QCM sensor disk was performed, using MIP based on the polymer consisting of acrylic acid, acrylamide, N-benzylacrylamide. Interestingly, only a pentadecapeptide (the linear epitope of the dengue virus NS_1_ protein) was used as template for MIP formation and direct whole NS_1_ detection. Purified and unpurified dengue NS1 proteins showed high affinity towards MIP with equilibrium dissociation constants (*K*_d_s) of 0.04 and 0.09 nmol L^−1^ [83]. The epitope imprinted MIP (EMIP) for the targeting of HSA was prepared from zinc acrylate, ethylene glycol dimethacrylate and adenosine triphosphate and successfully applied in the design of QCM-based biosensors. The prepared EMIP-QCM sensor was employed for the analysis of the real human serum sample. The LOD of the EMIP-QCM sensor was calculated to be 0.026 µg mL^−1^. The linear range of the HSA sensor measured from 0.050 g mL^−1^ to 0.500 g mL^−1^ and the recovery of real samples was in the range of 99.3% to 103 % [83]. A biomimetic sensor for the detection of human immunodeficiency virus type 1 glycoprotein gp41 was developed based on EMIP. A synthetic peptide with 35 amino acid residues was imprinted onto polydopamine and the recognition of the protein, gp41, was investigated using QCM. High affinity of formed MIP towards the imprinted peptide and specific binding of gp41 was proofed by calculated dissociation constant (*K*_d_ = 3.17 nmol L^−1^) [11]. 

### 5.3. Complementary SE/QCM-D Technique for Polymer Analysis and Biosensing

**Figure 5:**
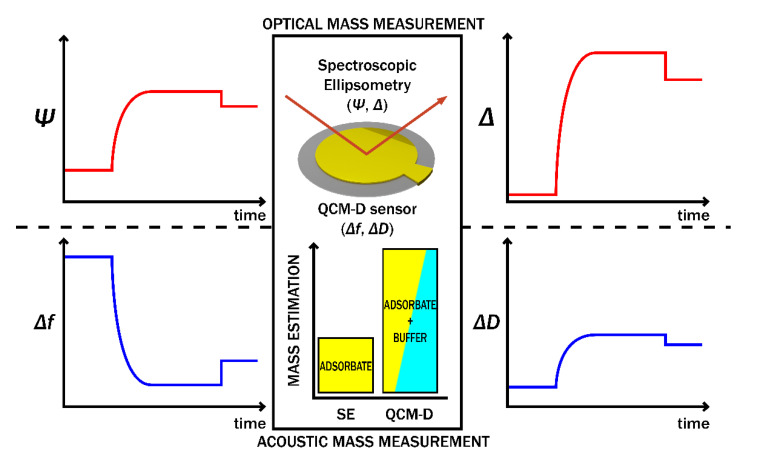
Schematic representation of combined SE/QCM-D technique used for the measurements. *Ψ*–ellipsometric parameter that corresponds to the light amplitude ratio upon reflection, Δ–ellipsometric parameter that corresponds to light wave phase shift, Δ*f*–time depended change in frequency, Δ*D*–time depended change in energy dissipation.

The combination of different sensing methods in the simultaneous measurement of one analyte attracted great interest due to the possibility of monitoring the signal of the same process using independent techniques [121]. Such a combination provides possibilities to evaluate processes occurring on the surface in real time more deeply and precisely. When SE and QCM-D are complementary in one system, it is possible to apply both techniques simultaneously and derive the solvent content of very thin adsorbed polymer layers on the surface [96]. These surface-sensitive methods can provide information on swelling processes and adsorption mechanisms of various materials, such as proteins [14,98]. The schematic representation of the combinatorial SE/QCM-D measurement technique is presented in Figure 5. 

The formation from dopamine methacrylamide copolymerized with 2-hydroxyethyl methacrytate and glycidyl methacrylate was investigated using SE and QCM-D techniques. The results demonstrated that this terpolymer can be applied for antibody immobilization on a surface suitable for biosensing applications. The immobilization of anti-aflatoxin M1 antibody, using a concentration of 0.08 mg mL^−1^ on the surface of a silicon wafer, spin coated with terpolymer, was measured through in situ SE and QCM-D. The increase of polymer film thickness measured using SE was 7 nm, thus indicating antibody adsorption on the surface. The calculated surface mass density from the ellipsometric data of the adsorbed antibody measured 3.5 mg m^−2^. This value was similar to findings of other authors when a monolayer of antibodies was formed. In addition, the process of antibody immobilization was investigated by QCM-D. The shift in resonant frequency from −40 to −60 Hz and dissipation shifts from 2 × 10^−6^ to 9 × 10^−6^ were obtained. Combining SE/QCM-D data analysis provided consistent evidence for the formation of the anti-aflatoxin antibody monolayer [93]. The complementary technique of SE/QCM-D was suggested as an appropriate candidate for biosensing applications. The complementary QCM-D and generalized ellipsometry (GE) was applied for the characterization and investigation of the swelling characteristics of the PAA and the PNIPAAm polymer brushes and adsorption of the BSA using in situ dynamic mode. The QCM-D/GE analysis of the adsorption process of BSA revealed that BSA adsorbs to PAA under attractive electrostatic conditions. When the BSA solution was injected, an increase in thickness from 5 to 22 nm was observed due to the adsorption of BSA on the surface of the PAA brushes. QCM-D data analysis showed the increase in a real mass up to approximately 38 mg∙m^−2^ [95]. The combinatorial method of GE/QCM-D was demonstrated as suitable for the real-time, non-invasive characterization of protein adsorption to these complex polymer coatings.

## 6. Conclusions and Future Trends

Recent advances in the development and application of spectroscopic ellipsometry (SE) illustrate that this method is well suited for real-time investigation of various polymeric structures, ranging from synthetic polymers without or with biomolecules (protein, DNA, etc.) to highly complex polymer-based composites (such as protein-modified layers used for biosensing purposes). Various advantages of SE such as rapid analysis, high precision and sensitivity, and nondestructive nature exhibit the great potential of this method for future applications in detailed polymer analysis, particularly in fields where polymers are applied in the design and fabrication of organic electronics and optical devices, including biosensors. As ellipsometry can be applied not only for the investigation of optical properties but also for the thickness of layers, it can be widely applied for different kinds of polymers. This renders SE a powerful universal analytical tool. Special attention should be paid in the future for investigation of the optical properties of various polymer brushes using SE, as such brushes bear high feasibility in biosensor applications. Moreover, the application of SE in the TIR mode represents a very promising approach and could be used in combination with electrochemical methods similarly to electrochromic optical recording. 

Quartz crystal microbalance with dissipation (QCM-D) enables evaluation of various properties of polymeric layers including swelling, viscosity, and elasticity of these polymers. Moreover, QCM-D enables instant registration of an analytical signal based on variations at fundamental frequency and at several harmonics, which provides excellent opportunities to assess numerous physical/chemical properties of polymers, including shear-forces, hardness, swelling, hydration, etc. However, the ability to exploit QCM-D patterns registered at several harmonics remain not well understood and hence they are rarely practically exploited. The detailed analysis of dissipation at different harmonics based on the mathematical modelling could provide new insights into the mechanical properties of polymers formed on the surface of the QCM-D chip and/or interaction between biomolecules, one of which should be immobilized on the QCM-D chip. Therefore, we expect significant experimental and theoretical research activity regarding the application of QCM-D in fundamental investigations of polymeric layers and assessment of affinity interactions between proteins, DNA complementary strands and some other biomolecules. It is remarkable that recent experimental setups enable us to apply a combination of spectroscopic ellipsometry with quartz crystal microbalance with dissipation (SE/QCM-D) techniques, which opens new horizons for the evaluation of the polymeric structures and interactions between biomolecules.

## Figures and Tables

**Table 1:** The summary of SE and QCM-D application for the assessment of polymer layers and for the application in biosensing.

Techniques	Polymer	Parameters Studied	Application	Ref.
**SE**	Zeonor	Optical properties	Detection of hCG using sandwich immunoassay format	[76]
	Polyacrylamides	Layer morphology and thickness dependance on concentrations of dsDNA and incubation time	For dsDNA immobilization	[77]
	Plasma polymer	Refractive index, porosity, thickness	Evaluation of enzymes BLC and HRP stability	[78]
	Nitrogen-containing plasma polymer	Ellipsometric parameter *Ψ* dependance on the wavelength, layer thickness.	HRP covalent binding capacity and interaction with anti-HRP antibodies	[79]
	Polymer brushes containing poly(N-isopropylacrylamide) and poly(acrylic acid)	Polydispersity, grafting density, thickness in dry and wet states	HSA and FIB—adsorbing and repelling properties of nanometric thickness polymer brushes at various temperatures and pHs	[66]
**QCM or QCM-D**	Acrylamide-based MIPs (polyacrylamide, N-hydroxymethylacrylamide, N-isopropylacrylamide)	Rebinding capacity, relative imprinting factors, selective adsorption and recognition properties	Detection of BHb	[80]
	Acrylic acid—N-vinylpyrrolidone—N,N′-(1,2-dihydroxyethylene) bis-acrylamide based MIP	QCM response (Δ*f*, Hz) to different concentrations of D1R	Biosensors for D1R detection (LOD = 4.3 μmol L^−1^, LOQ = 5.9 μmol L^−1^); MIP distinguish between the free receptor, receptor-dopamine complexes, and receptor-antagonist complexes	[81]
	Multilayer films of poly(acrylic acid), poly(allyamine hydrochloride) and silica nanoparticles	QCM response (Δ*f*, Hz) during the formation of multilayers, effect of pH and nanoparticles size, crosslinking	Absorption of BSA	[82]
	acrylic acid, acrylamide, N-benzylacrylamide epitope-mediated MIP	QCM response (Δ*f*, Hz) for different analyte concentrations	Recognition of NS1	[83]
	zinc acrylate, ethylene glycol dimethacrylate based MIP	QCM response (Δ*f*, Hz) for different concentrations of analyte,	Biosensor for HSA determination (LOD = 0.026 µg mL^−1^)	[84]
	polydopamine epitope-mediated MIP	QCM response (Δ*f*, Hz) to different concentrations of analyte	Biosensor for HIV-1 gp41 determination (LOD = 2 ng mL^−1^)	[11]
	Polypyrrole	QCM response (Δ*f*, Hz) to different concentrations of analyte	Immunosensor for anti-HSA determination (LOD = 0.01 mg mL^−1^) Immunosensor for anti-PRV determination from diluted serum samples.	[85]
	Polypyrrole	QCM response (Δ*f*, Hz) during polymer formation, calculation of surface mass dencity	Evaluation of enzymatic synthesis of Ppy layer using immobilized GOx, evaluation of enzyme activity	[86]
	Polypyrrole	QCM response (Δ*f*, Hz) during the electrochemical Ppy synthesis	Evaluation of electrochemical formation of aggregated Ppy particle based layer	[87]
	Polypyrrole based MIP	QCM response (Δ*f*, Hz) during electrochemical formation of MIP-Ppy layer.	Biosensor for caffeine detection	[10]
	Polyaniline	QCM response (Δ*f*, Hz) during electrochemical synthesis of PANI and proteins adsorption	The effect of the PANI film thickness and used doping agents on adsoption of BSA and FIB	[88]
	PEDOT bearing sialic acid-terminated trisaccharides	QCM response (Δ*f*, Hz) during polymer layers formation and binding of analyte	Specific recognition of H1N1 (*K*_D (app.)_ = 0.96 HAU on the 2,6- sialyllactose- modified surfaces; LOD = 0.12 HAU)	[89]
	Methacrylic acid–vinylpyrrolidone–dihydroxyethylene bisacrylamide based MIP	QCM response (Δ*f*, Hz) for MIP and NIP characterization and for the analyte detection	Sensor for quantitative determination of Hev b1 (LOD = 1 μg L^−1^)	[90]
	Mixed-charge poly-L-lysine with anionic oligopeptide side-chains	QCM-D response (Δ*f*, Hz and Δ*D*, 10^−6^) for the polymer antifouling properties analysis	BSA adsorption on the polymer surface	[91]
	Poly-L-lysine	QCM-D response (Δ*f*, Hz and Δ*D*, 10^−6^) during PLL formation, gold nanoparticles and antibody fragments immobilization, abd analyte detection	Immunosensor for BLV gp51 antigen detection	[92]
	DMA, HEMA, GMA copolymers	SE (polymer dry thickness, in-situ polymer swelling, antibody immobilization measurements), QCM (antibody immobilization measurements)	IgG antibody immobilization on the polymer surface	[93]
**Complementary SE/QCM-D**	Carboxylated poly(oligoethylene glycol-co-2-hydroxymethyl methacrylate)	SE (film thickness, polymer characterization). QCM response (Δ*f*, Hz) for the polymer modification with rabbit IgG and interaction with anti-rabbit IgG	Biosensors for goat anti-rabbit IgG recognition	[94]
	Poly-(*N*-isopropylacrylamide)	Complementary GE/QCM-D analysis (layer porosity and thickness measurements).	BSA adsorbtion to the polymer surface.	[95]
	Poly-(acrylic acid)	Complementary SE/QCM-D Polymer layer swelling in response to temperature, areal mass and viscosity measurements.	pH responsive retention and release of BSA from polymer surface.	[96]
	Polypyrrole	QCM response (Δ*f*, Hz), SE response (*Ψ*,Δ) during PPy film growth with varying GOx concentrations.	PPy-GOx modified electrode. Manufacturing of electrochemical glucose sensor	[97]
	Poly(2-vinylpyridine)	Complementary SE/QCM-D in-situ analysis of Pd ion and Pd-NP enriched polymer brushes (thickness, mass, viscosity, shear modulus, proportion of metal)	Application in catalytically active nanocoatings	[98]
	Polymer brushes from PNIPAM, PMEO_2_MA, PDMA, POEGMA, PHEMA	QCM-D (Δ*f*, Hz and Δ*D*, 10^−6^) and SE (Ψ,Δ, thickness, refractive index) response during polymer brush swelling in response to temperature.	Surface and volume hydrofilicity determination for polymer brushes as a function of temperature. Explanation of PHEMA brush antifouling properties. Further application to hydrogels.	[99]
	PDADMAC, CHI, JR 400	QCM-D and SE thickness measurements during polycation adsorption onto negatively charged surfaces, effects of the polymer concentration, charge density, chemical nature, ionic strength of the solution and the addition of a surfactant, hydration level	Electrostatically-driven enhanced polymer. deposition	[100]

Human chorionic gonadotrophin (hCG), double stranded DNA (dsDNA), bovine liver catalase (BLC), horseradish peroxidase (HRP), anti-horseradish peroxidase (anti-HRP), glucose oxidase (GOx), human serum albumin (HSA), bovine hemoglobin (BHb), dopamine D1 receptor (D1R), bovine serum albumin (BSA), Dengue virus protein (NS1), HIV-1 glycoprotein gp41 (HIV-1 gp41), poly(pyrrole) (PPy), poly(ortho-phenylenediamine) (PoPD), fibrinogen (FIB), human influenza virus (H1N1), Poly(3,4-ethylenedioxythiophene) (PEDOT), hemagglutinating units (HAU), rubber latex allergens (Hev b1), bovine leukemia virus gp51 antigen (gp51), pseudorabies virus antibodies (anti-PRV), Immunoglobulin G (IgG), dopamine methacrylamide (DMA), 2-hydroxyethyl methacrylate (HEMA), glycidyl methacrylate (GMA), poly(2-vinylpyridine) (P2VP), poly(N-isopropylacrylamide) (PNIPAM), poly(di(methoxyethoxy)ethyl methacrylate) (PMEO_2_MA), poly(N,N-dimethylacrylamide) (PDMA), poly(oligo(ethylene glycol) methacrylate) (POEGMA), poly(2-hydroxyethylmethacrylate) (PHEMA), poly-L-lysine (PLL), poly-(diallyldimethylammonium chloride) (PDADMAC), chitosan (CHI), hydroxyethylcellulose quaternized with 2,3-epoxypropyltrimethylammonium chloride (JR 400), Δ*D* energy dissipation.

## Data Availability

The data presented in this study are available on request from the corresponding author.
